# Assessing Extreme Seed Longevity: The Value of Historic Botanical Collections to Modern Research

**DOI:** 10.3389/fpls.2019.01181

**Published:** 2019-10-14

**Authors:** Gareth Porteous, Mark Nesbitt, Jonathan P. Kendon, Christina J. Prychid, Wolfgang Stuppy, Maria Conejero, Daniel Ballesteros

**Affiliations:** ^1^Science Directorate, Royal Botanic Gardens, Kew, London, United Kingdom; ^2^School of Biological and Chemical Sciences, Queen Mary University of London, London, United Kingdom; ^3^Botanic Garden, Ruhr-University Bochum, Bochum, Germany

**Keywords:** historic collections, botanical collections, differential scanning calorimetry (DSC), *in vitro* culture, seed germination, transmission electron microscopy, aging, ancient seeds

## Abstract

Botanical, historical, and archaeological collections have been the source of extraordinarily long-lived seeds, which have been used to revive extinct genotypes or species. The longest-lived example of a viable seed of known age is the date palm, *Phoenix dactylifera* L., of which an estimated 2000-year-old seed was germinated in 2005. Seed longevity is important for agriculture and biodiversity conservation, and understanding the basis for the extraordinary longevity of seeds from botanical collections could help improve seed banking technology. In this work, we studied the viability and structural features of date palm seeds collected in Baghdad in 1873 and stored in the Economic Botany Collection (EBC) at the Royal Botanic Gardens, Kew, and seeds collected in 2004 and stored dry at −20°C in the Millennium Seed Bank (MSB). Viability was studied by attempted seed germination and *in vitro* culture of embryos, and structural features were studied by X-rays, transmission electron microscopy, and differential scanning calorimetry. We found that the seeds preserved in the MSB did not decrease in viability, with ultrastructural features similar to those in freshly harvested seeds. In contrast, the 144-year-old seeds were dead, and large ultrastructural changes were observed, particularly in the storage lipids (size, distribution, and melting properties) and other storage constituents. These results contrast with previous reports that date seeds could remain viable for ∼2000 years in uncontrolled storage environments. We did not find that the postharvest treatment of the EBC seeds in the 19th century, or their storage conditions at Kew, was more deleterious than that which was likely encountered by the ∼2000-year-old seeds. These results highlight the role of well-documented collections in establishing whether reports of extraordinary longevity are ordinarily repeatable.

## Introduction

Reliably dated seeds from historic collections have shown extraordinary longevity. Seeds of the genera *Leucospermum*, *Liparia*, and *Acacia* collected in the early 19th century in the Cape region, South Africa, were germinated after 218 to 270 years of suboptimal storage, developing into plants of typical appearance (Daws et al., 2007). Similarly, viability in seeds up to 211 years old was reported from seven species extracted from adobe brick buildings of California and Northern Mexico (Spira and Wagner, 1983). In addition, there are further reports of germination of seeds from diverse species, dated between 90 and 144 years old (Bewley and Black, 1982; Bowles et al., 1993; Steiner and Ruckenbauer, 1995; Godefroid et al., 2011). Old sediments have also been the source of seeds and plant tissues that have shown extraordinary longevity. For example, several *Nelumbo nucifera* seeds dated between 200 and 1288 years old were found alive in old lotus fruits preserved in a Holocene dry lake in northeastern China (Shen-Miller, 2002; Shen-Miller et al., 2002). Archaeological seeds have shown some of the greatest longevity. For example, a viable seed of *Canna compacta* was removed from a ceremonial rattle necklace in a tomb in Argentina, presumed to be c. 600 years old (Bewley and Black, 1982). However, the record for the oldest mature seed that has been recovered alive belongs to the date palm *Phoenix dactylifera* (Sallon et al., 2008). A 2000-year-old seed recovered in the excavations of Masada, a Herodian fortress overlooking the Dead Sea, was germinated and grown and the seedling named “Methuselah” after the long-lived biblical figure.

Reviving historic seeds, in addition to bringing seed science to the attention of the general public, could bring to life extinct cultivars and genotypes that might be useful to understand crop domestication and evolution, or even improve current crops (Sallon et al., 2008). In addition, old seeds found in herbaria and historic botanical collections could be used (and have been used) to generate seedlings for population reinforcement of rare species (i.e. increasing population size by adding plant individuals to a still existing population) or even to revive species that were extinct in the wild (Bowles et al., 1993; Godefroid et al., 2011). Furthermore, these seeds could bring important knowledge on the fundamental basis of seed longevity, resilience, and stress tolerance (Shen-Miller, 2002). Seed longevity is important for agriculture and biodiversity conservation (e.g. Walters et al., 2005; Li and Pritchard, 2009; Walters et al., 2010; Sano et al., 2015), and understanding the basis for the extraordinary longevity of seeds located in herbaria, historical, or other botanical collections could offer knowledge that may be applied to the improvement of the current seed banking technology or help to extend the longevity of short-lived seeds (Shen-Miller, 2002). While reports of extraordinary long-lived seeds may be exceptional cases (Shen-Miller et al., 2002; Sallon et al., 2008), they could also represent the potential longevity of a species due to specific structural or biochemical characteristics (Shen-Miller, 2002; Daws et al., 2007), and perhaps further examples of extraordinary longevity can be found in other collections.

In 1875, Joseph Dalton Hooker, director of the Royal Botanic Gardens, Kew, communicated to the Linnean Society of London the observations of William Henry Colvill (1833–1885), a Scottish surgeon-major of HM Indian Forces and Civil-Surgeon (Colvill, 1875). In this, Colvill described his collection of diverse date palm fruits, made during an expedition in the Province of Baghdad in 1873, which were soon after sent to Kew and acknowledged in Kew’s annual progress report (Hooker, 1873). After arriving there in December 1873, the date fruits were stored in one of the buildings of the former Museum of Economic Botany and then transferred in about 1988 to the purpose-built Sir Joseph Banks Building as part of the Economic Botany Collection (EBC). Given that the seeds of date palm are potentially very long-lived (Sallon et al., 2008) and that seeds within the date palm fruits of Colvill’s collection have been stored dry and at typical room temperatures since arriving at Kew, we thought it possible that these seeds were still viable.

The present study compares this historic collection reference material of date palm seeds (*P. dactylifera* L.) from the EBC to modern seeds held at the Millennium Seed Bank (MSB), with the aim of comparing physiological, histological, ultrastructural, and physiochemical characteristics. Seed structure and function are intimately related (e.g. McNeil et al., 1984; Bouman and Boesewinkel, 1995; Purkrtova et al., 2008; Walters et al., 2010), so we expect that ultrastructure would be highly maintained in long-lived seeds after storage, particularly when they have preserved their viability through time. The present study investigates ultrastructure in two states of the date palm seed: “dry” (i.e. as dry as they are in the relevant collection store) and “imbibed” (i.e. after seeds were fully imbibed in water). “Dry” seeds (e.g. at the MSB conditions) are likely to be in a solid or glassy state at which most metabolic activities are inhibited (Walters et al., 2010). Conversely, “imbibed” seeds will be in a fluid state, in which metabolic and repair activities, or programmed cell death (PCD) mechanisms promoted by aging can be present (Sano et al., 2015). This comparison between “dry” and “imbibed” seeds is also important because deterioration of seeds is more evident in the imbibed state than in the dry state (García de Castro and Martinez-Honduvilla, 1984; Xia et al., 2015). Date palm seeds possess “orthodox” storage physiology and so can be dried to lower than typical moisture content with minimal damage, often increasing longevity when combined with reductions in temperature (Bewley and Black, 1982; Royal Botanic Gardens Kew, 2019). Seeds of this species are also suitable for study because embryonic development has been comprehensively studied in this and related species (DeMason, 1984; DeMason, 1988; Sekhar and DeMason, 1989; Orozco-Segovia et al., 2003), providing a good starting point for understanding changes that occur in date seeds during storage.

The main aims of the present study are therefore to (1) study seed or embryo viability of experimental seed lots (particularly of the historic seeds stored at the EBC); (2) compare diverse structural features of the seeds stored in different collections (i.e. seeds of different ages); (3) analyze whether ultrastructural changes in the seeds occur mainly in the dry state, or after imbibition; and (4) determine the most likely causes of any deterioration observed. In addition, this work aims to emphasize the importance of historical collections in contextualizing observations of extreme seed longevity.

## Material and Methods

### Plant Materials

Modern date palm seeds (*P. dactylifera* L.) were obtained from the MSB where they had been stored since 2004, following standard seed bank protocols (dried at 15% relative humidity [RH] and stored at −20°C; Food and Agriculture Organization, 2014). These seeds were collected in the region of Balqa, Jordan, on September 22, 2003 (in a location <50 km from Masada, Israel, across the Dead Sea).

**Figure 1:**
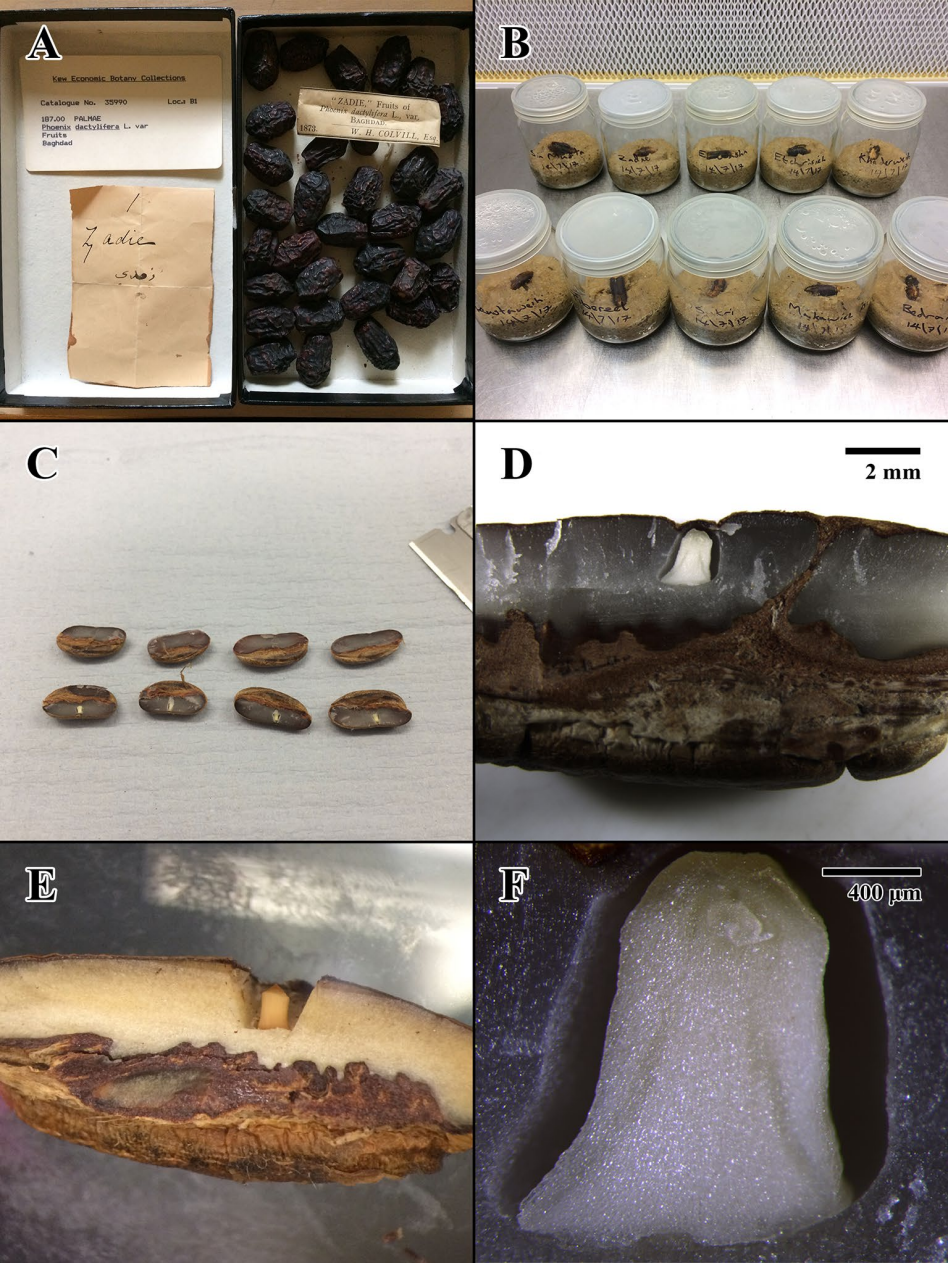
Photographs of Colvill’s original Economic Botany Collection (EBC) accession, germination tests, and physical dissection of seed lots. **(A)** 1873 fruits of EBC accession 35990 (“Zadie”) prior to dissection. **(B)** Start of germination test for EBC seeds in sterile conditions, prior to placement in incubator. **(C)** Physical dissection of 2004 seed lot (Millennium Seed Bank [MSB] accession no. 211945). **(D)** Dissection close-up of 2004 MSB seed. Scale bar = 2 mm. **(E)** Dissection close-up of 1873 EBC seed. **(F)** Compound microscope dissection close-up of 2004 MSB seed. Scale bar = 400 μm.

Historic date palm fruits were obtained from the EBC at Kew. These fruits were recorded in the Museum Entry Book as being accessioned at Kew on December 19, 1873, having been collected in Baghdad and sent to Kew sometime in September 1873 (Colvill, 1875). They would have been stored in glass-topped boxes or (more likely) in sealed glass jars. In about 1988, the fruits were transferred to acid-free card boxes (**Figure 1A**) and moved to a purpose-built store in the Sir Joseph Banks building at Kew. Environmental conditions in the former museum buildings are not recorded, but these are sturdy brick buildings, and thus, indoor conditions will not have been extreme. Conditions in the Joseph Banks building are maintained at approximately 16°C and 40 to 55% RH, with a period of several years at 11°C around the year 2000.

A total of 29 seeds from 13 different EBC accessions were used for the diverse experiments of this study. One to four seeds were obtained from each accession, with each landrace or cultivar bearing vernacular names as recorded by W. H. Colvill in 1873 (Colvill, 1875): EBC catalog number 35988 (“Drah Subbah”), 35990 (“Zadie”), 35991 (“Brem”), 35992 (“Makawieh Eshgar”), 35993 (“Etchrisieh”), 36006 (“Tiburzel”), 36007 (“Sin Mufta”), 36008 (“Khaderawieh”), 36011 (“Makawieh Ahmur”), 36012 (“El Washa”), 36021 (“Sukri”), 36024 (“Bedraieh”), 36025 (“Khustawieh”) (Colvill, 1875). The seeds were extracted by hand from the EBC fruits for the purposes of this study. A longitudinal incision was made into the fruit to remove the seed, and the flesh was retained in the EBC.

As historic germplasm was very limited, we could not use standard technical replicates of the 144-year-old seeds from each of the EBC accessions in the different experiments performed. Instead, to show variation across ancient seeds, we used 3 to 10 randomly selected seed samples for each experiment, each coming from one of the different EBC accessions (summarized in **Table 1**). Moreover, to have variation across individual accessions, we used two to four seeds of each of the accessions sampled in two to four of the different experiments used to test seed viability or structural integrity. With this experimental design, we minimized any negative impact on the EBC collection but at the same time added repeatability of the results across experiments and across accessions.

**Table 1:** Seeds from the Economic Botany Collection (EBC) used for the different experiments performed in this article.

EBC catalog number	Vernacular names (Colvill, 1875)	Experiment	Number of seeds used
35988	Drah Subbah	DSC	2
35990	Zadie	X-ray/germination Light microscopy/TEM (dry)	1 1
		*In vitro* culture	1
35991	Brem	DSC	1
35992	Makawieh Eshgar	DSC	1
35993	Etchrisieh	X-ray/germination Light microscopy/TEM (imbibed)	1 1
36006	Tiburzel	X-ray/germination	1
		Light microscopy/TEM (dry)	1
36007	Sin Mufta	X-ray/germination Light microscopy/TEM (dry)	1 1
36008	Khaderawieh	X-ray/germination	1
		Light microscopy/TEM (dry) *In vitro* culture	1 1
		DSC	1
36011	Makawieh Ahmur	X-ray/germination Light microscopy/TEM (imbibed)	1 1
36012	El Washa	X-ray/germination	1
		Light microscopy/TEM (imbibed) DSC	1 1
36021	Sukri	X-ray/germination Light microscopy/TEM (dry)	1 1
36024	Bedraieh	X-ray/germination Light microscopy/TEM (imbibed)	1 1
36025	Khustawieh	X-ray/germination Light microscopy/TEM (imbibed)	1 1
		*In vitro* culture	1

### X-Ray Test

The X-ray test is a noninvasive and nondamaging test that is typically used at the start of seed germination experiments to check that seeds are not empty or infested (Bruggink and Van Duijn, 2017). This test also confirms that the seeds have developed and maintained gross morphological features necessary to germinate, with no obvious signs of deterioration, which may hamper viability. A total of 10 seeds from 10 different collections of the 1873 seed lot (**Table 1**) were X-rayed to check the percentage of full seeds in this historic collection and to determine embryonic position and structural status as intact and potentially healthy (i.e. “full”). In addition, three seeds of the 2004 seed lot (MSB accession no. 211945) were scanned to obtain images for comparison. The X-ray test was conducted using an Ultrafocus Faxitron X-ray machine and the Vision NDT version 2.4.1U software (Faxitron Bioptics LLC, Tucson, Arizona).

### Germination

Germination tests were used to check the germination rate and estimate the viability of experimental seed samples included in this study. For MSB samples, we used data from the most recent test carried out on March 13, 2015, based on standard operating protocols developed by the MSB (Davies et al., 2015). In this test, 50 seeds were placed in two clear large plastic boxes (200 × 120 × 60 mm) each with 500 g of 1,000 μm^2^ graded sieved sand, mixed with 75 ml of distilled water [amount based on previous experiments (De Vitis et al., 2014)], and 25 seeds per box were sown on the surface of the sand medium, with the operculum in contact with the substrate to permit imbibition. Original data for germination of the seeds stored in the MSB since 2004 is available in the internal MSB database (MSB accession no. 211945). We used these values (instead of generating new germination data), as access to additional germplasm of this accession was restricted due to low quantities remaining in storage at the MSB (<1,000 seeds). Seeds were stored in standard seed bank conditions, and the germination score of this accession was assumed to have not changed in the period 2015–2017.

The ten 1873 seeds that received X-ray test pretreatment were also used to assess germination percentage. X-rays offer a nondestructive method that is believed not to affect seed germination and viability (Simak and Gustafsson, 1953; Bino et al., 1993; Bruggink and Van Duijn, 2017). A surface sterilization step was added to the main seed germination protocol to reduce the risk of fungal and bacterial contamination of the historic seeds (Merritt et al., 2007). Seeds were soaked in water for 2 min and mildly surface sterilized for 2 min in 0.5% (w/v) sodium dichloroisocyanurate with a drop of Tween 20. After rinsing in sterile deionized water, the seeds were placed individually into 10 autoclaved 75-mm-diameter glass jars each with 75g of 1,000 μm^2^ graded sieved sand mixed with 15 ml of distilled water. All seeds were sown on the surface of the sand medium with the operculum in contact with the substrate to allow imbibition (**Figure 1B**).

In all cases, seeds were stored in an incubator with a 12/12-h (light/dark) photoperiod at 30°C constant temperature, and germination results were scored on a weekly basis using a seed germination scorecard. We used these germination conditions as they are the optimal germination conditions assayed for this species following the standard seed bank operating protocols developed by the MSB (Davies et al., 2015). Sallon et al., 2008, applied gibberellic acid (GA_3_) and other plant hormones and fertilizers prior to potting the seeds 1 cm deep in sterile potting soil. We decided not to use plant hormones in this initial test, as standard germination conditions previously proved enough to germinate more than 200-year-old seeds (Daws et al., 2007), and the entire date palm seeds lack the dormancy that needs to be broken by the use of GA_3_. Instead, *in vitro* plant tissue culture protocols for embryo rescue were used to determine embryo viability, as explained below.

Viability for all seed lots was estimated by analyzing germination results and, upon completion, using a cut test to assess nongerminated seeds as fresh, moldy, empty, or infested (Davies et al., 2015). A tetrazolium test was used to confirm results using accepted protocols (Peters, 2005).

### *In Vitro* Culture of Embryos

In addition to germination by standard seed bank protocols, three additional EBC seeds were selected for embryo rescue and *in vitro* culture (EBC accession nos. 35990, 36008, 36025). As per the 1873 seeds used for germination tests and microscopy analysis, a longitudinal incision was made into the fruit to remove the seed, and the flesh was retained in the EBC. A fresh date of the “Medjool” cultivated variety was shop-bought on the day of *in vitro* culture initiation and subjected to identical treatments to act as a positive control for the following procedure.

Seeds were surface-sterilized according to Sarasan et al. (2002). Following rinsing in sterile deionized water, the seeds were dried on a sterile filter paper in the laminar air flow. Using a flame-sterilized stainless-steel SEM sectioning blade, the seeds were cut open longitudinally, taking care not to damage the embryo. As an additional antibacterial step, the embryos were placed in a solution of 50% (v/v) Plant Preservative Mixture^™^ (PPM^™^) for 1 min, followed by 2 min of air drying.

Embryos were placed on Murashige and Skoog (MS) medium including vitamins (Murashige and Skoog, 1962), supplemented with 3% (w/v) sucrose, 0.3% (w/v) activated charcoal, and 0.8% (w/v) agar. PPM^™^ was added at a concentration of 3 ml L^−1^ (Sarasan et al., 2006) to control endophytic contamination, and 28.9 µM GA_3_ was added to stimulate germination. Cultures were incubated at 30°C at 12/12-h photoperiod under cool white fluorescent light (PPFD 25µmol m^−2^ s^−1^). Embryos were left for 12 weeks on this medium to germinate.

In the event of no germination after 8 weeks, embryos were transferred to three different media to stimulate production of somatic embryos or callus from any remaining viable cells. This media consisted of MS medium (Murashige and Skoog, 1962) supplemented with 3% (w/v) sucrose, 0.3% (w/v) activated charcoal, and 0.8% (w/v) agar and the following plant growth regulators: (1) 24.6 µM indole-3-butyric acid (IBA); (2) 24.6 µM IBA plus 4.55 µM thidiazuron (TDZ); (3) 45.5 µM 2,4-dichlorophenoxyacetic acid (2,4-D) plus 13.65 µM TDZ. Cultures were incubated at 30°C in darkness.

### Desiccation, Storage Conditions, and Imbibition of the Seeds

Seed histology and ultrastructure were studied in dry seeds and seeds after imbibition. A total of 10 seeds from the 2004 seed lot (MSB accession no. 211945) and 10 seeds of the 1873 seed lot (**Table 1**) were studied using comparative analysis. Five seeds from 2004 and five seeds from 1873 (EBC accession nos. 35990, 36006, 36007, 36008, 36021) were maintained in a dry state (at or close to respective storage humidity) to investigate cellular ultrastructure during reduced moisture storage conditions, while the other five seeds from 2004 and five seeds from 1873 (EBC accession nos. 35993, 36011, 36012, 36024, 36025) were imbibed to study cellular changes occurring in a wet state. The imbibed seeds were placed on five sheets of filter paper in a glass Petri dish (25 × 150 mm) each containing 30 ml of deionized water, for 48 h. This method was based on previous studies to control water uptake and avoid imbibition injury (Tilden and West, 1985). Prior to imbibition, all seeds were equalized at room temperature for at least 72 h. Where appropriate, the RH (RH%) of each seed lot was confirmed using a calibrated Rotronic HygroPalm and Rotronic AwDC-DIO hygrometer probe (Rotronic AG, Bassersdorf).

### Seed Embryo Excision for Microscopy

Embryo excisions were made by cutting open the date palm seeds with a razor blade and gently applying force with a 0.5 kg bronze weight (**Figure 1C**). The primary incision was made longitudinally adjacent to the operculum on the underside of the seed to avoid damage to the embryo, which is located in a small cavity inside the endosperm (**Figures 1D–F**). The resulting embryos were carefully removed using tweezers and immediately transferred into fixative.

### Microscopy

Light microscopy and transmission electron microscopy (TEM) were used for comparative analysis of the dry and imbibed (wet state) seeds. Five embryos per each seed lot (1873, 2004) and moisture pretreatment (dry, imbibed) were prepared for microscopy observations. Embryos were fixed in Karnovsky solution (2% paraformaldehyde and 2.5% glutaraldehyde in 0.05 M phosphate buffer, pH 7.2) (Glauert, 1975) for a minimum period of 12 h at 5°C. Samples were buffer-washed, postfixed with 1% osmium tetroxide solution for 2 h, buffer-washed again, and then dehydrated through a graded ethanol series, prior to a slow (4 days) infiltration through a graded ethanol: LR white resin series. Individual embryos were placed in sectioning AGAR molds (5 × 12 × 3 mm) with 100% resin and the resin polymerized in a Fistreem vacuum oven at 60°C for 24 h at 300 mbar pressure. Resin-embedded tissue samples were cut into sections of appropriate thickness using a Reichert-Jung Ultracut microtome. (Leica Microsystems; Wetzlar, Germany)

Samples for light microscopy were sectioned at 0.5-μm thickness with a Reichert Ultramicrotome, using glass knives produced by Leica EM KMR3 and LKB 7800A (Leica Microsystems; Wetzlar, Germany) knife makers. Sections were stained using 0.5% toluidine blue in 0.1 M phosphate buffer, pH 7.0 (Feder and O’Brien, 1968), observed and photographed on a Leica LMD7 digital light microscope with Leica DFC7000T digital camera attachment.

Samples for TEM were sectioned with a Reichert Ultramicrotome between 0.05 and 0.1 μm using a DiATOME ultra 45º (serial no. MF368) diamond knife. Ultrathin sections were collected on formvar-coated copper slot grids and then poststained with uranyl acetate and lead citrate.

All sections (light microscopy and TEM) were cut at the height of the epicotyl by pinpointing the embryonic pore (using 0.5-μm semithin sections to find this tissue region). The embryonic pore is a distinct anatomic feature of the date palm cotyledon, conserved at the level of the epicotyl, through which the first leaf emerges during late germination (DeMason and Thomson, 1981). This confirmed the precise location for all transverse semithin and ultrathin tissue sections, enabling high-accuracy image-based comparative analysis between differing cellular tissues (cotyledon parenchyma, epicotyl), sample treatments (dry, imbibed), and sample ages (2004, 1873). Prepared grids were viewed on a Hitachi H-7650 transmission electron microscope (Tokyo, Japan) and imaged with the integral AMT XR41 digital camera.

### Differential Scanning Calorimetry

Thermal behavior of historic and recent seeds was measured in seed embryos using differential scanning calorimetry (DSC). Four seeds were scanned from the MSB accession and six seeds from EBC accessions (**Table 1**). All seeds were scanned at the moisture conditions at which they were stored in the different collections, which were 0.040 ± 0.005 g H_2_O/g dry weight (DW) and 0.113 ± 0.008 g H_2_O/g DW for the MSB and EBC seeds, respectively. However, three of the EBC seeds were dried for 1 month at 15°C and 15% RH before the DSC and achieved 0.049 ± 0.004 g H_2_O/g DW. Three fresh dates of the “Medjool” variety that were shop-bought were also scanned after drying to 0.041 ± 0.006 g H_2_O/g DW for comparison purposes.

This experiment was conducted to investigate change in the melting properties of the storage lipids [i.e. triacylglycerols (TAGs)] of seed embryos. Seed deterioration has been shown to affect the physical properties of lipids by decreasing the energy of the melting transition of TAG (Vertucci, 1992), and this measurement enables us to further characterize the physical and structural status of the seeds. Differential scanning calorimetry scans were also used to determine the glass transition temperature (Tg) of the seeds stored at the particular storage condition in both (MSB and EBC) collections. The Tg is useful in understanding whether seeds were stored in a solid (i.e. glassy) state at which most biochemical and cellular reactions are inhibited (i.e. below the Tg) or seeds were at a fluid state at which biochemical and cellular reactions are possible (i.e. above the Tg) (Walters et al., 2010).

Phase transitions in embryos were determined using a Mettler-Toledo DSC-1 (Greifensee, Switzerland), calibrated for temperature with indium (156.6°C) standards and for energy with indium (28.54 J g^−1^). The presence of phase transitions was determined from heating thermograms recorded between −150°C and +90°C while scanning at a rate of 10°C min^−1^. The onset temperature of the melting transitions was determined from the intersection between the baseline and a line drawn from the steepest portion of the transition peak. The enthalpy (ΔH) of the transition was determined from the area encompassed by the peak and the baseline. Second-order transitions in heating scans were identified as glass melting events, and the Tg was assigned as the midpoint in the displacement of power during the scan (e.g. Ballesteros et al., 2017). All analyses were performed using Mettler-Toledo Stare software version 12.0, Mettler-Toledo, Switzerland. Enthalpies of exothermic and endothermic events are expressed on a per g DW basis.

## Results

### X-Ray

**Figure 2:**
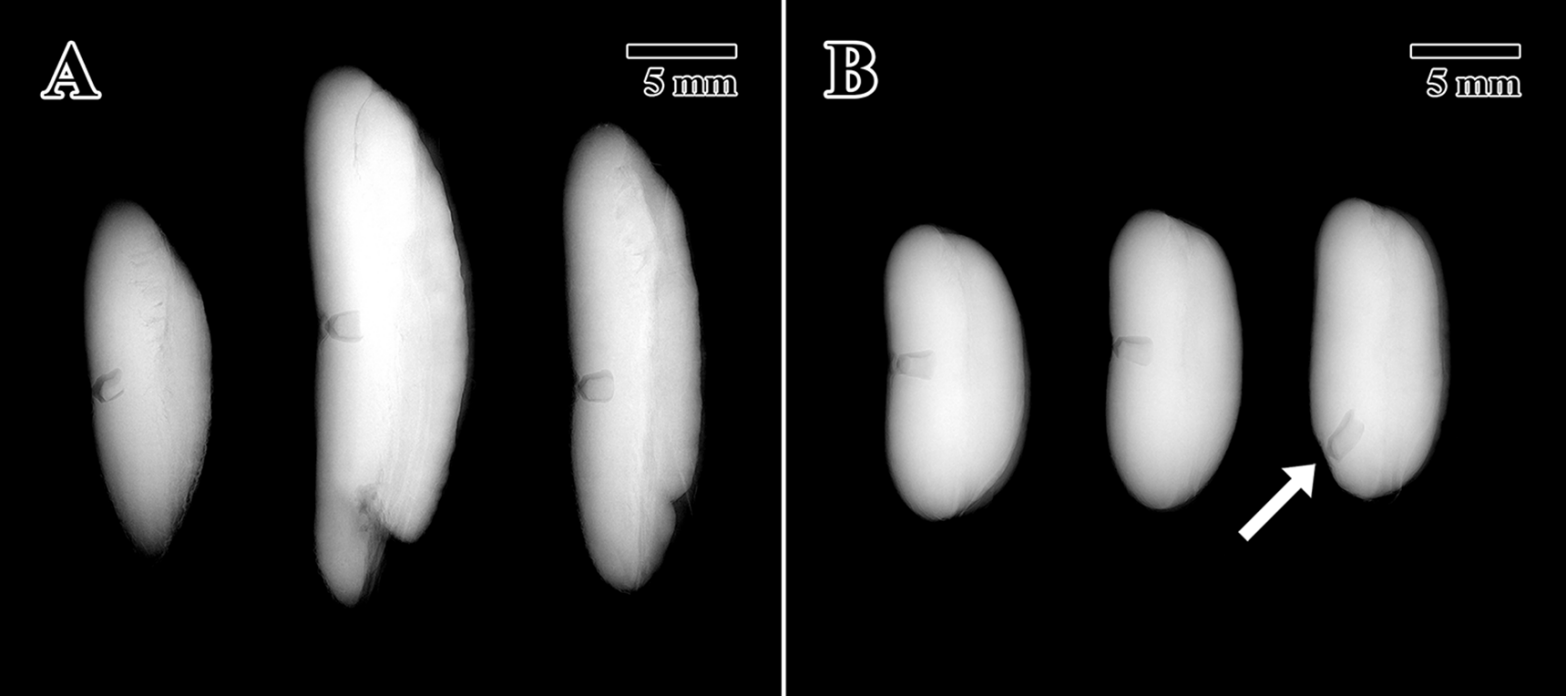
X-ray tomogram of experimental seed lots. **(A)** 1873 EBC seed lot sample. **(B)** 2004 MSB seed lot sample. (Arrow = embryo location at distal portion of the endosperm). Scale bar = 5 mm.

X-ray tests indicated that all 10 seeds (100%) scanned from the 1873 seed lot presented full intact embryos (**Figure 2A**). For the 2004 seed lot only, three seeds were scanned after confirming that the percentage of full seeds was 100% in a previous scan on October 14, 2004 (unpublished data). All seeds tested showed full and highly contrasted embryos located in the center of the endosperm (**Figure 2B**), except for a single 2004 seed where the embryo was found to have developed toward a distal end of the endosperm instead of the standard position towards the center of the seed (arrow in **Figure 2B**).

### Germination

The 2004 seed lot (MSB accession no. 211945) showed 88% germination with mean time to germination (T50) of 27.4 days. These seeds showed 100% viability after a cut test was performed on nongerminated seeds (i.e. nongerminated seeds were full, white, hard, and healthy, showing no signs of being moldy, empty, or infested). The 1873 seed lot showed no germination over the entire duration of the germination test performed on MSB seeds (63 days), and no germination was found after an extension of the germination test for a further 18 months to ensure enough time was used for this historical germplasm (as old seed tend to germinate slowly). In the cut test performed after the long germination test, the seed embryo did not show obvious signs of being moldy, empty, or infested, but it was yellow, soft, and mushy, indicating that embryo tissues were dead. A subsequent tetrazolium test confirmed there were no viable cells in any of the embryos. Due to 0% germination in the 1873 seed lot, the condition of the embryo seen after the cut test, and the negative result in the tetrazolium test, 0% viability was accepted for this historic germplasm.

### *In Vitro* Culture of Embryos

Embryos excised from the control “Medjool” variety seeds began to germinate (i.e. root elongation) after 2 weeks in culture and by 8 weeks had produced a first leaf. None of the embryos from the 1873 seed lot germinated in the 8-week period, and so they were transferred to medium containing plant growth regulators. Twelve weeks following transfer of the embryos to medium-containing plant growth regulators, no growth was observed, leading to the conclusion that none of the embryos’ cells were viable.

### Light Microscopy

**Figure 3:**
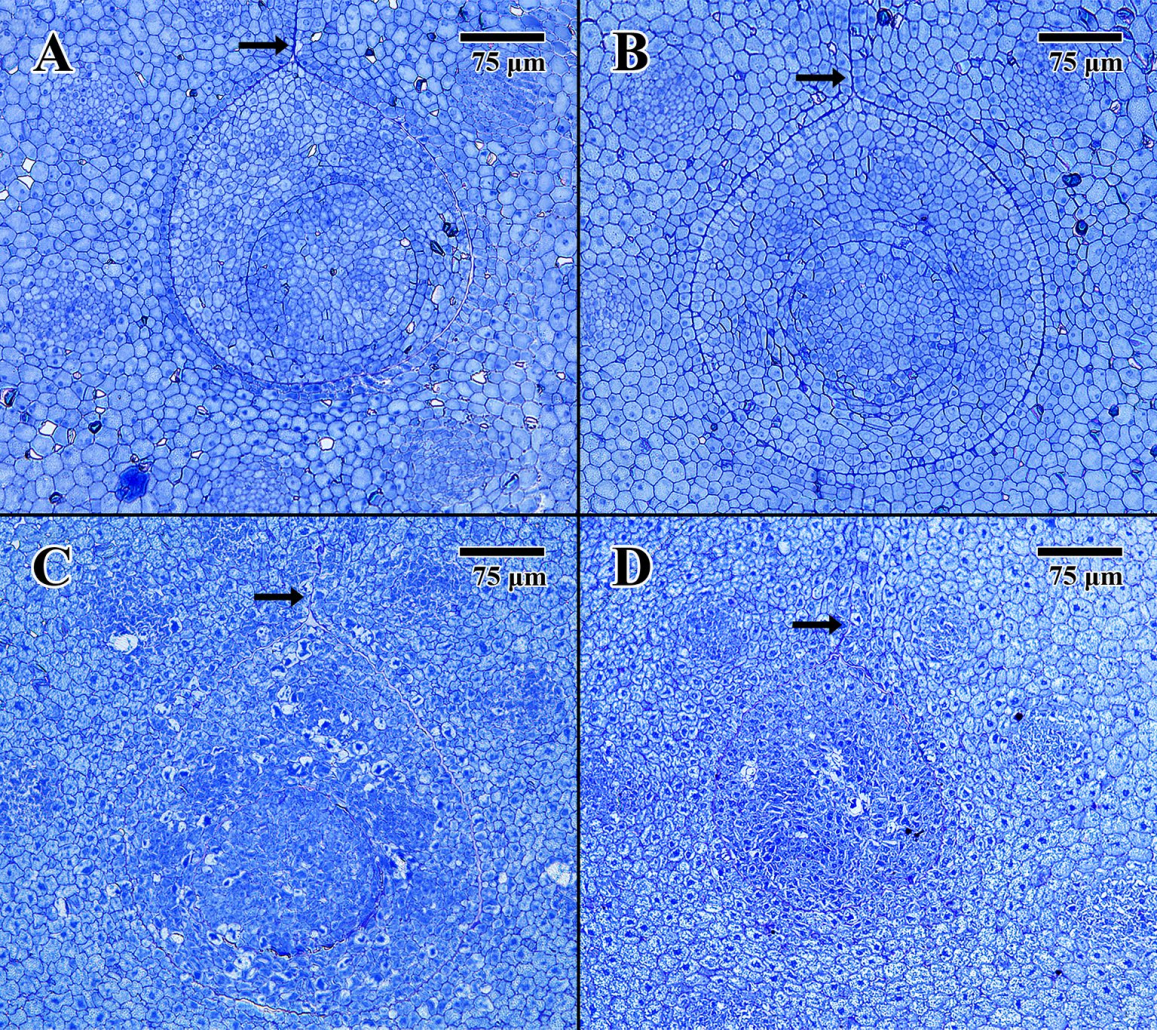
Light microscope micrographs cut 0.5 μm thick at the location of the embryonic pore. **(A)** Transverse section of 2004 embryo in dry state (MSB accession no. 211945). **(B)** Transverse section of 2004 embryo in imbibed state (MSB accession no. 211945). **(C)** Transverse section of 1873 embryo in dry state (EBC accession no. 36007). **(D)** Transverse section of 1873 embryo in imbibed state (EBC accession no. 36011). (Arrows = pore aperture.) Scale bar = 75 μm.

Light microscope histology captured images of the epicotyl region surrounded by less-differentiated cotyledon parenchyma. The pore aperture through the cotyledon (arrows in **Figure 3**) confirms cellular tissue in all cases is at the precise location of the epicotyl (DeMason and Thomson, 1981).

The dry 2004 embryo (**Figure 3A**) and the imbibed 2004 embryo (**Figure 3B**) appear well structured, showing clear areas of provascular tissue within both the surrounding cotyledon and central epicotyl (outer circle). The epicotyl in both cases is bounded by a uniform array of protoderm and contains what is thought to be an early-stage leaf primordium (inner circle).

The dry 1873 embryo (**Figure 3C**) and the imbibed 1873 embryo (**Figure 3D**) both appear to be composed of many collapsed cells, with none of the cells having normal shape or turgidity. The provascular tissue looks highly denatured, and the lack of staining in some cells, especially within the epicotyl of **Figure 3C**, indicates that these cells are likely to be empty of constituents or highly vacuolated.

### Electron Microscopy

**Figure 4:**
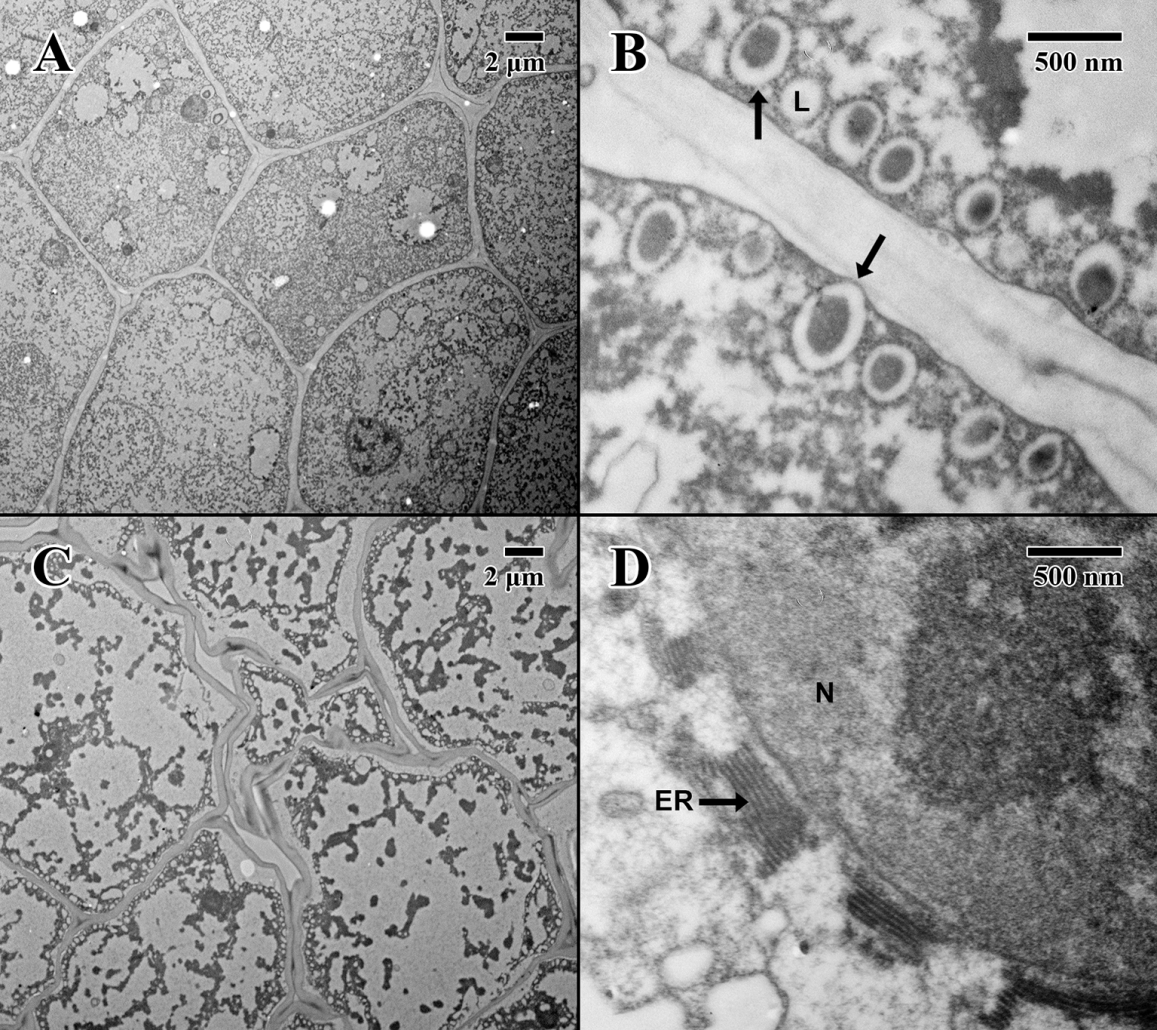
Transmission electron microscope micrographs of cotyledon parenchyma in date palm embryos in dry state. **(A)** ×1,000 2004 embryo (MSB accession no. 211945). Scale bar = 2 μm. **(B)** ×10,000 2004 embryo (MSB accession no. 211945). Scale bar = 500 nm. **(C)** ×1,000 1873 embryo (EBC accession no. 36007). Scale bar = 2 μm. **(D)** ×10,000 1873 embryo (EBC accession no. 36007). Scale bar = 500 nm. (N, nucleus; L, lipid body; ER, endoplasmic reticulum; arrows = explained in text).

The cotyledon parenchyma (dry state) of a 2004 embryo (**Figure 4A**) is uniform in shape, with a densely stained cytoplasm containing protein storage vacuoles. At higher magnification (**Figure 4B**), the plasma membrane is pulled away from the cell wall, and lipid bodies line the edges of cells with dense dark-stained regions of condensed material (arrows in **Figure 4A**). In contrast, the cotyledon parenchyma (dry state) of an 1873 embryo (**Figure 4C**) appears angular and poorly structured, with large gaps between cells. Few nuclei are present, and the cytoplasm appears mostly empty. Lipid bodies line the edges of cell membranes, but appear white and irregular. Some cells show significant signs of degraded cytoplasm, observed near endoplasmic reticulum adjacent to just a few cell nuclei (arrow in **Figure 4D**).

**Figure 5:**
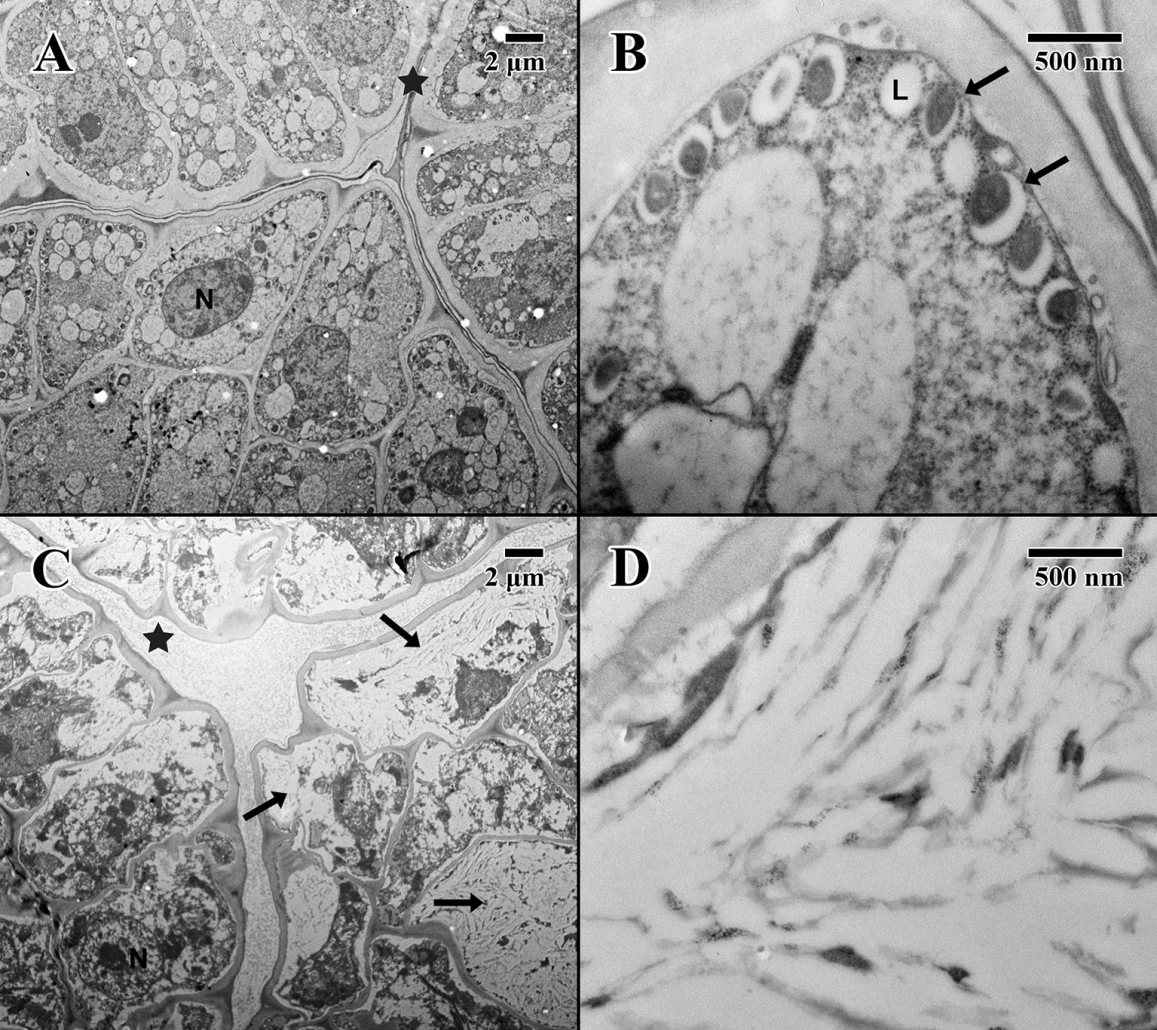
Transmission electron microscope micrographs of the epicotyl region of date palm embryos in dry state. **(A)** ×1,000 2004 embryo (MSB accession no. 211945). Scale bar = 2 μm. **(B)** ×10,000 2004 embryo (MSB accession no. 211945). Scale bar = 500 nm. **(C)** ×1,000 1873 embryo (EBC accession no. 36007). Scale bar = 2 μm. **(D)** ×10,000 1873 embryo (EBC accession no. 36007). Scale bar = 500 nm. (N, nucleus; L, lipid body; Star = embryonic pore, arrows = explained in text.

The epicotyl region (dry state) of a 2004 embryo (**Figure 5A**) at the precise location of the embryonic pore (star in **Figure 5A**) is rich in nuclei and organelles. At higher magnification (**Figure 5B**), lipid bodies are numerous, and most possess dense dark stained constituents, as seen in the parenchyma (arrows in **Figure 5B**). Numerous cytoplasmic ribosomes are attached to the circumference of the lipid bodies, suggesting cells are in a “resting” state. In comparison, within the epicotyl region (dry state) of an 1873 embryo (**Figure 5C**) at the precise location of the embryonic pore (star in **Figure 5C**), cells appear to be highly degraded and suffering loss of cellular contents (arrows in **Figure 5C**), where much of the organellular ultrastructure is unrecognizable apart from denatured nuclei. At higher magnification (**Figure 5D**), large white areas of the cytoplasm appear to be extremely damaged with irregular patterned remains.

**Figure 6:**
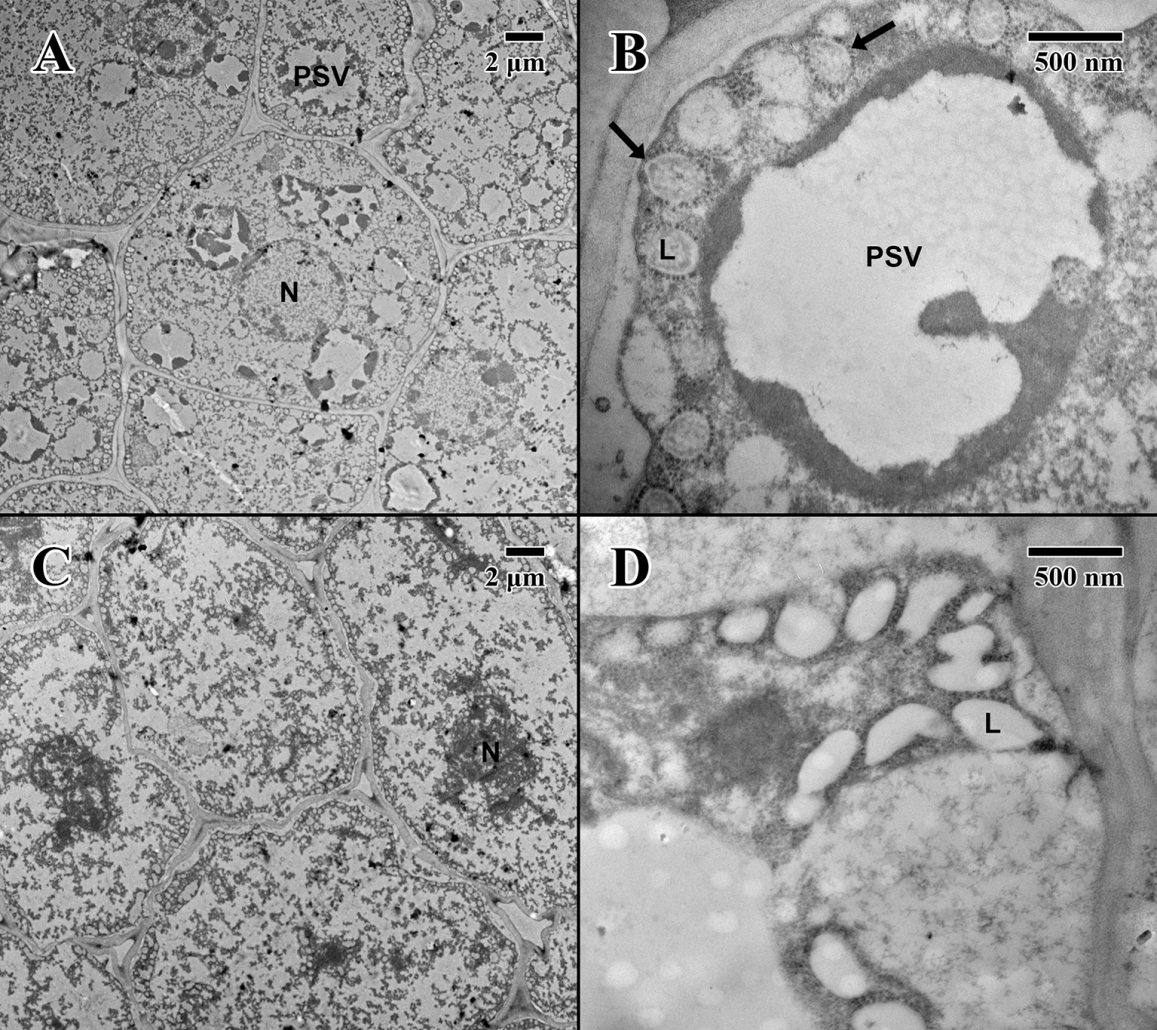
Transmission electron microscope micrographs of cotyledon parenchyma of date palm embryos in the imbibed state. **(A)** ×1,000 2004 embryo (MSB accession no. 211945). Scale bar = 2 μm. **(B)** ×10,000 2004 embryo (MSB accession no. 211945). Scale bar = 500 nm. **(C)** ×1,000 1873 embryo (EBC accession no. 36011). Scale bar = 2 μm. **(D)** ×10,000 1873 embryo (EBC accession no. 36011). Scale bar = 500 nm. (N, nucleus; L, lipid body; PSV, protein storage vacuole; arrows = explained in text.)

The cotyledon parenchyma (imbibed state) of a 2004 embryo (**Figure 6A**) contains abundant nuclei and swollen protein storage vacuoles. At higher magnification (**Figure 6B**), lipid bodies are light gray and granular (arrows in **Figure 6B**). Protein storage vacuoles contain a dark irregularly shaped mass thought to be phytin (DeMason and Thomson, 1981)—the major source of calcium, magnesium, and phosphorous in seeds, utilized in seedling development (Bewley and Black, 1978). Comparatively, the cotyledon parenchyma (imbibed state) of an 1873 embryo (**Figure 6C**) is angular, and cellular structure appears to have been compromised with few nuclei or organelles. At higher magnification (**Figure 6D**), lipid bodies appear irregular in shape and appear to have broken and merged (arrows in **Figure 6D**).

**Figure 7:**
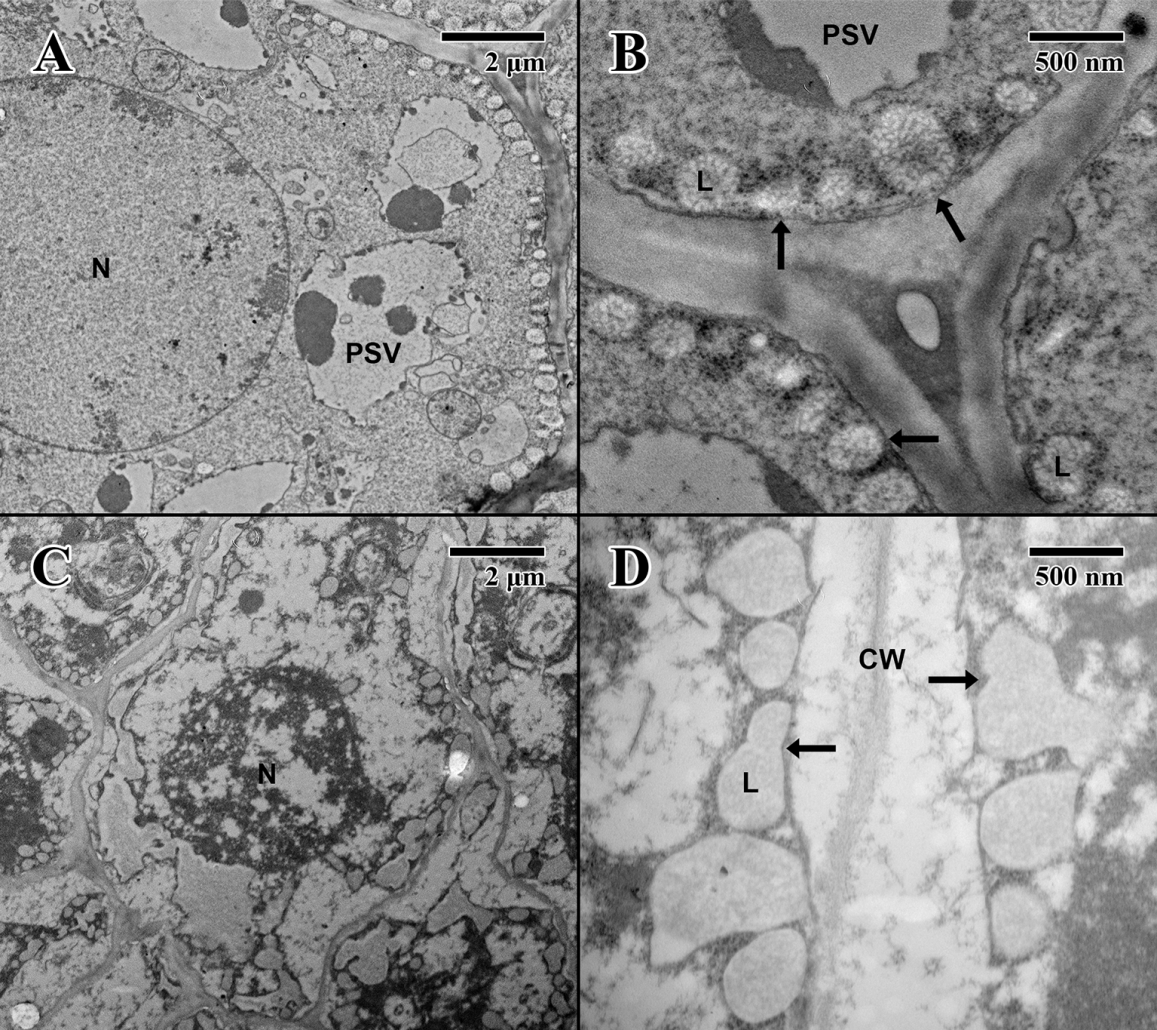
Transmission electron microscope micrographs of epicotyl region of date palm embryos in imbibed state. **(A)** ×2,500 2004 embryo. Scale bar = 2 μm. **(B)** 10,000 2004 embryo. Scale bar = 500 nm. **(C)** ×2,500 1873 embryo (EBC accession no. 36011). Scale bar = 2 μm. **(D)** ×10,000 1873 embryo (EBC accession no. 36011). Scale bar = 500 nm. (N, nucleus; CW, cell wall; L, lipid body; PSV, protein storage vacuole; arrows = explained in text.).

The epicotyl region (imbibed state) of a 2004 embryo (**Figure 7A**) at the height of the embryonic pore (not shown) possesses cells with large nuclei and protein storage vacuoles. At higher magnification (**Figure 7B**), lipid bodies line the edges of cell membranes, and like the imbibed cotyledon parenchyma (**Figure 6B**), they appear light gray and granular, with abundant cytoplasmic ribosomes present—indicative of cellular activity (arrows in **Figure 7B**). On the other hand, within the epicotyl (imbibed state) of an 1873 embryo (**Figure 7C**) at the height of the embryonic pore (not shown), cells appear to be suffering high degrees of lysis and chronic loss of cellular contents. At higher magnification, it is apparent that the plasma membrane has been retracted, and many lipid bodies appear to have ruptured and fused (arrows in **Figure 7D**).

### Thermal Behavior of Date Embryos

**Figure 8:**
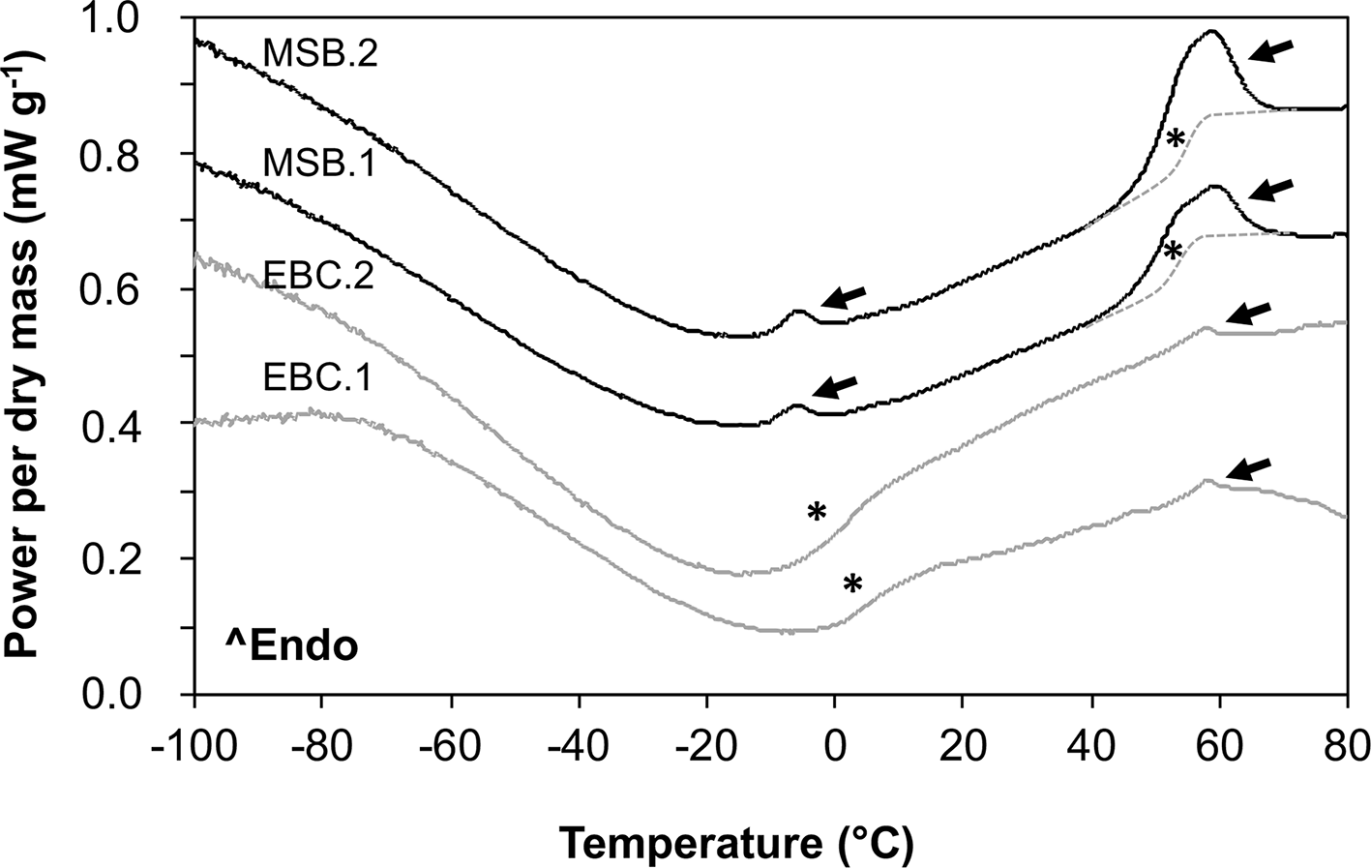
Melting scans of embryos from *Phoenix dactylifera* seeds stored for 13 years in the MSB or for 144 years in the EBC of the Royal Botanic Gardens, Kew. Embryos were scanned at a rate of 10°C/min in a differential scanning calorimeter. Arrows indicate first-order melting transitions that are related to the melting of the triacylglycerols (TAG) in the storage lipids. Asterisks indicate a step change in the baseline that is related to the glass transition temperature (Tg). The Tg is masked by the TAG melting between 40°C and 65°C (they both occur at the same temperature range), and a line has been drawn as a guide for the eye. EBC1 and EBC2 correspond to samples obtained from one seed from accessions 35988 and 35992, respectively. MSB1 and MSB2 correspond to samples obtained from two different seeds in the accession 211945.

In MSB stored seeds (**Figure 8**), as well as in fresh “Medjool” dates (scans not shown), two clear endothermic events were detected in heating scans as peaks around −5°C and +55°C. These peaks were identified as TAG melting transitions (after Ballesteros and Walters, 2007) based on the low amount of water in the embryo tissues (**Figure 8** shows date embryos dried to about 0.04 g H_2_O g DW^−1^, TAG melt is indicated by black arrows). At about −5°C, embryos from seeds stored in the MSB showed a small TAG enthalpy of melting that averaged 0.6 ± 0.2 J g DW^−1^; however, this TAG melting transition was absent in historic seeds of the EBC (**Figure 8**). At about +55°C, the TAG melting transitions were larger in the MSB stored seeds (enthalpy of melting averaged 8.6 ± 1.9 J g DW^−1^) than historic seeds (enthalpy of melting averaged 0.9 ± 0.2 J g DW^−1^). Temperatures of the peaks of these TAG melting transitions (53°C ± 10°C and 57°C ± 2°C for MSB and EBC seeds, respectively, **Figure 8**) were not significantly different in a *t* test (*P* > 0.05). The Tgs were detected at 54.5°C ± 5.5°C and 58.2°C ± 1.4°C in the dry seeds of the MSB and the EBC (scanned at 0.040 ± 0.005 and 0.049 ± 0.004 H_2_O g DW^−1^, respectively). In contrast, seeds stored under the EBC moisture conditions (0.113 ± 0.008 H_2_O g DW^−1^) showed the Tg at 1.8°C ± 1.6°C.

## Discussion

Our experiments found that the 144-year-old historic date seeds studied had no viability (i.e. no seed germination or embryo growth *in vitro*). In addition, the ultrastructure of the cells of the embryo (in both the “dry” and “imbibed” state) showed major disruptions in the cytoplasm and membrane integrity of lipid bodies with clear signs of lysis or autophagy. Major disruptions in lipid bodies are typically observed in aged seeds (García de Castro and Martinez-Honduvilla, 1984; Dawidowicz-Grzegorzewska and Podstolski, 1992; Fotouo-M et al., 2015; Xia et al., 2015), and lysis or autophagy is typical in the last phases of PCD, which is one of the results of seed deterioration during aging (El-Maarouf-Bouteau et al., 2011; Kumar et al., 2015; Sano et al., 2015). Furthermore, the low enthalpy of melting in the storage lipids is indicative of large seed deterioration (cf. Vertucci, 1992). These results contrast with our expectations based on previous reports that date seeds could remain viable during ∼2,000 years or more in uncontrolled storage environments (Sallon et al., 2008).

The major structural disruptions in the EBC seeds in both the “dry” and “imbibed” state indicate that the ultrastructural changes observed occurred mainly during the “dry” state in which seeds were stored after harvest, likely as a consequence of the seed aging process. Economic Botany Collection seed embryos had relatively high moisture content compared to seeds stored in the MSB (averaged 0.113 and 0.040 g H_2_O/g DW for EBC and MSB, respectively), and this is reflected in the Tg difference observed between EBC and MSB stored seeds (c. 55°C and 2°C, respectively). Based on the Tg of EBC seeds, the seed embryos in the EBC collection have not been preserved in the glassy state at which the major metabolic and cellular reactions that lead to seed aging are inhibited (Walters et al., 2010), while those in the MSB were. Thus, it is probable that “dry” seeds in the EBC were in a fluid-like state and were slowly aging until clear ultrastructural signs of PCD appeared (Sano et al., 2015). These large ultrastructural changes are also observed in seeds exposed to the warm and moist conditions used in seed accelerated aging experiments (at which seeds are also in a fluid-like state) or in seeds aged in uncontrolled storage conditions (usually at 75–100% RH and 35–65°C) (García de Castro and Martinez-Honduvilla, 1984; Dawidowicz-Grzegorzewska and Podstolski, 1992; Fotouo-M et al., 2015; Xia et al., 2015). Conversely, ultrastructural changes tend to be minor if “dry” seeds are stored at moisture and temperature conditions in which a glassy state may be favored (e.g. Xia et al., 2015).

The differences in viability and longevity between previous reports of ∼2000-year-old date palm seed germination (Sallon et al., 2008) and the EBC 144-year-old seeds may be related to differences in storage conditions. Dry and cold environments promoting the glassy state would have extended seed longevity, while moist and hot environments promoting a fluid state would have decreased seed longevity (Li and Pritchard, 2009; Walters et al., 2010; Sano et al., 2015). It has been speculated that “high summer temperatures and low precipitation at Masada” (Sallon et al., 2008), where the ∼2000-year-old seeds were found, contributed to exceptional longevity by minimizing the associated degenerative effects of free radical production (Sallon et al., 2008). However, RH at Masada, Israel, ranged between 4% and 100% over the last 35 years, averaging 56%, 42% RH at noon (the driest hour of the day) and 62% RH at 6 am (the most humid hour of the day) (Israel Meteorological Service, 2019). Indeed, in ∼82% of the days, the lowest RH was >30% (60% of the days RH was between 30% and 50%), and in ∼81% of the nights, the highest RH was >50% (24% of the nights, RH was >70%) (Israel Meteorological Service, 2019). This data confirms the Masada site is not exceptionally dry for seed storage and conservation. If this was the case, it would have been expected to be <30% RH for most of the time (optimal RH for seed storage is between 10% and 25%; The Food and Agriculture Organization, 2014). In addition, it is also likely that these seeds were exposed to daily temperatures fluctuations between 17°C and 30°C with extremes of 0°C and 47°C (Israel Meteorological Service, 2019). With such conditions, seeds at Masada would have been stored mostly above the Tg over the 2000-year period [based on DSC scans and the fact that the Tg/moisture relationship is a highly conserved trait among seeded plant species (Sun and Leopold, 1997)]. Consequently, this suggests that the Masada seeds may have been stored in a state in which active or potentially degenerative biochemical and cellular reactions were possible over the long storage duration.

The EBC 144-year-old seeds were collected in Baghdad during 1873, before September of that year (Colvill, 1875), shipped to the UK between September 1, 1873, and December 19, 1873, stored in glass-topped boxes or sealed glass jars in the relatively uncontrolled conditions of the museum for 115 years, and transferred to acid-free card boxes at about 16°C and 45% to 55% RH for 29 more years. Environmental conditions in the museum where they were stored for 115 years are not known, but indoor conditions will not have been extreme. It is likely that temperatures in the museum remained <25°C [e.g. indoor temperatures of British and US museums in the early 20th century used to remain between 13°C and 25°C (Brown and Rose, 1996), and indoor temperatures in British houses averaged from 3°C to 16°C in the late 1960s and early 1970s (Mavrogianni et al., 2013; Vadodaria et al., 2014)] and RH <70% [e.g. RH in British museums in the late 19th century and early 20th century tended to average 50% to 60%, ranging between 10% and 30% in winter to 60% and 90% in summer (Brown and Rose, 1996); the EBC date fruits were not affected by mold, which usually grows when RH >70% in nutrient-rich media such as dates (Broström and Larsen, 2015)]. As observed in the DSC scans, these conditions favored the storage of seeds above the Tg over the 144-year period with the aging consequences discussed above. However, taking into account that the differences in storage conditions between the 2000-year-old dates from Sallon et al. (2008) (averaged temperature and RH between 10% and 30°C and 42% and 62%, respectively) and the 144-year-old dates (averaged temperature and RH between 13% and 25°C and 50% and 60%, respectively) were likely small, and the fact that seeds in both places may have been stored above the Tg, it is unlikely that the difference in storage conditions explains why 33% (i.e. one out of three seeds plants) of the 2000-year-old seeds tested in Sallon et al. (2008) germinated, while none of the 23 EBC seeds did. With such storage conditions, we should expect seed deterioration to occur at a comparable rate.

Postharvest procedures and storage of the 144-year-old dates from the EBC before they were shipped to the UK in 1873 could have also affected their deterioration and unexpected short longevity. For example, when dates are harvested before they dry in the tree (in the stage known as Khalaal), it is common to boil them for at least 20 to 30 min before drying as a preservation method (Barreveld, 1993). While boiling dates is a great preservation method for the fruit, it will kill the seeds and their embryos (boiling seeds for 5 min is used in some protocols to provide a population of dead seeds, e.g. Marrero et al., 2007). Khalaal dates are bright yellow or red, compared to darker colors in the dates that dry in the tree. Colvill (1875) states in his notes that 21 of 26 of the varieties shipped to Kew were “light” in color, and 5 were “dark.” While it could be possible that some of these light varieties were boiled Khalaal dates, Colvill carried out detailed investigations into date husbandry and would have mentioned such a procedure. Equally, it is unlikely that all date varieties were harvested in the Khalaal stage, as only two or three varieties are sweet enough to be harvested in such a stage (Barreveld, 1993). Poor date storage in Baghdad or during shipping (e.g. in extremely hot and moist environments) would have also contributed to seed deterioration. However, this is unlikely, as the time spent in transit would have been minimized, thanks to the opening of the Suez Canal in 1869 and relatively swift access to the Mediterranean [used extensively by British vessels and significantly reducing previous travel distance and time to London by >40% in some cases (Rabino, 1887)]. Additionally, the dates collected by Colvill were those handled and stored for human consumption, and extreme environments would have been avoided. In other material presented to the British Museum by his uncle Dr William Sharpey FRS (secretary of the Royal Society 1853–1872 and correspondent of Charles Darwin), Colvill was praised for the excellent state of his collection from Baghdad (Günther, 1874). Furthermore, Colvill was recognized for making over 100 successful contributions to Kew’s collections over this period (Royal Botanic Gardens, Kew, 1901). In addition, any dates exposed to hostile environments in transit would have been attacked by mold, and no signs of fungal growth were found in the EBC dates used (e.g. **Figure 1A**). Finally, as a medical professional, Colvill was very detailed in his notes, and none of the damaging postharvest procedures indicated above are mentioned in his meticulous records.

Other notable studies investigating the longevity of date seeds (Spira and Wagner, 1983), tested 183-year-old materials, but none of the 27 seeds tested were deemed viable. Considering the multiple factors of this study, the full results from our varied experiments indicate limited evidence for the extreme longevity of *P. dactylifera* seeds. From our results in this work, we conclude that the use of historic botanical collections for assessing claims of extreme seed longevity offers valuable additions to modern research, highlighting the role of well-documented collections in establishing whether reports of extraordinary longevity are ordinarily repeatable. However, analysis can prove more complicated than it initially appears. There is a delicate balance to be struck between using reports of revived ancient germplasm as a guide as to which historic collections should be further tested for viability and the destruction of finite collection material to assess seed longevity at any cost.

## Data Availability Statement

The datasets generated for this study are available on request to the corresponding author.

## Author Contributions

WS conceived the study; WS, DB, CP, and GP contributed to the design of the study; MN assisted acquisition of historic material; GP and DB assisted acquisition of modern material; GP performed light microscopy and TEM, helped and guided by CP and MC, who also provided laboratory support; JK performed *in vitro* culture and analysis; GP and JK performed seed germination tests; DB performed DSC and analyzed the data; DB and GP wrote the manuscript; MN and JK contributed to different sections of the final manuscript. All authors contributed to manuscript revision and read and approved the submitted version.

## Funding

This research was supported by funding granted by the Royal Botanic Gardens, Kew and Queen Mary University of London. The Royal Botanic Gardens, Kew, receives grant-in-aid from the Department for Environment, Food and Rural Affairs, UK.

## Conflict of Interest

During the first draft of the manuscript GP was a master’s degree student (September 2016–September 2017) and supervised by DB at the Royal Botanic Gardens, Kew, United Kingdom. The remaining authors declare that the research was conducted in the absence of any commercial or financial relationships that could be construed as a potential conflict of interest.
